# TAPaC—tobacco-associated particulate matter emissions inside a car cabin: establishment of a new measuring platform

**DOI:** 10.1186/s12995-022-00359-x

**Published:** 2022-08-24

**Authors:** Lukas Pitten, Dörthe Brüggmann, Janis Dröge, Markus Braun, David A. Groneberg

**Affiliations:** Institute of Occupational Medicine, Social Medicine and Environmental Medicine, Goethe University Frankfurt, Theodor-Stern-Kai 7, 60590 Frankfurt am Main, Germany

**Keywords:** Indoor air pollution, In-cabin PM concentration, Smoking in vehicles, Passive smoke, Environmental tobacco smoke, Second-hand smoke

## Abstract

**Background:**

Particulate matter (PM) emission caused by tobacco combustion leads to severe health burdens worldwide. Second-hand smoke exposure is extraordinarily high in enclosed spaces (e.g., indoor rooms, car cabins) and poses a particular threat to the health of vulnerable individuals (e.g., children, elderly, etc.). This study aimed to establish a new measuring platform and investigate PM emissions under four different ventilation conditions inside a car cabin without exposing any person to harmful tobacco smoke.

**Methods:**

PM concentrations were measured during the smoking of 3R4F reference cigarettes in a Mitsubishi Space Runner (interior volume 3.709 m^3^). The cigarettes were smoked with a machine, eliminating exposure of the researchers. Cigarettes were extinguished 4.5 min after ignition, and PM measurements continued until 10 min after ignition.

**Results:**

High mean PM concentrations were measured for cigarettes without ventilation after 4.5 min (PM_10_: 1150 µg/m^3^, PM_2.5_: 1132 µg/m^3^, PM_1_: 861.6 µg/m^3^) and after 10 min (PM_10_: 1608 µg/m^3^, PM_2.5_: 1583 µg/m^3^, PM_1_: 1133 µg/m^3^)**.** 3R4F smoked under conditions with turned on ventilation resulted in reduction of PM compared to those smoked without ventilation after 4.5 min (PM_10_:-47.5 to -58.4%, PM_2.5_:-47.2 to -58%, PM_1_:-39.6 to -50.2%) and after 10 min (PM_10_:-70.8 to -74.4%, PM_2.5_:-70.6 to -74.3%, PM_1_:-64.0 to -68.0%). Cigarettes smoked without ventilation generated high PM peaks at 4.5 min (PM_10_: 2207 µg/m^3^, PM_2.5_: 2166 µg/m^3^, PM_1_: 1421 µg/m^3^) and at 10 min (PM_10_: 1989 µg/m^3^, PM_2.5_: 1959 µg/m^3^, PM_1_: 1375 µg/m^3^). PM peaks of cigarettes smoked under different ventilation modes varied at 4.5 min (PM_10_: 630-845 µg/m^3^, PM_2.5_: 625-836 µg/m^3^, PM_1_: 543 - 693 µg/m^3^) and 10 min (PM_10_: 124 - 130 µg/m^3^, PM_2.5_: 124 - 129 µg/m^3^, PM_1_: 118 - 124 µg/m^3^).

**Conclusion:**

The new measuring platform provides a safer way for researchers to investigate PM emissions of cigarettes. These data are comparable to published research and show that smoking in a parked vehicle with the windows closed generates harmful PM emissions even when the vehicle ventilation is in operation. Future studies should be carried out using the new measuring platform investigating PM exposure and PM distribution of in-vehicle smoking under a wide range of conditions.

## Background

During the past decades, the knowledge about environmental air pollution and its threat to human health has increased significantly. Particulate matter (PM) emitted when a cigarette is smoked is highly carcinogenic. Tobacco smoke contains more than 5000 chemical substances, about 98 of which are proven to cause cancer, while many others are yet to be identified [1]. In addition, the toxic mixture of tobacco smoke affects multiple organ systems and leads to a large number of complications and diseases (e.g., cancer, asthma, etc.) [2, 3]. PM can be divided into PM_10_, PM_2.5_, and PM_1_. PM_10_ includes all particles with a size (aerodynamic diameter) ≤ 10 µm, PM_2.5_ includes all particles ≤ 2.5 µm, and PM_1_ ≤ 1 µm [4]. Smaller particles pose a greater threat to our health than relatively large particles as they can penetrate deeper into the lungs and get absorbed by the bloodstream, and reach the systemic circulation [5, 6]. Combustion sources (e.g., tobacco) primarily generate small particles [7]. The 2021 updated air quality guidelines of the World Health Organization (WHO) recommend a 24-h mean PM_2.5_ of ≤ 15 µg/m^3^, and a 24-h mean of 45 µg/m^3^ for PM_10_ [8]. According to Schramm et al., the sidestream smoke of cigarettes has a higher PM mass than mainstream smoke [9].

Although about 7 million tobacco smokers die per year because of their smoking, an estimated 1.2 million nonsmokers die of exposure to second-hand smoke (SHS) according to WHO data [10]. This is a dramatic number since the people affected by the associated burden are not actively deciding to smoke but are often exposed involuntarily and are at substantial risk for diseases such as chronic inflammatory diseases of the airways, asthma, chronic obstructive pulmonary disease, and lung or breast cancer [11]. As studies have shown, children are particularly susceptible to SHS. Due to their smaller lungs and higher respiratory rates, children inhale more particles per kg body weight than adults [12–14]. Pulmonary and respiratory diseases are significantly more common in children exposed to SHS than those not exposed [8, 13, 15]. To reduce the individual and economic burden caused by SHS, many countries have banned smoking in public places such as restaurants, cinemas, and bars [16–18]. Nevertheless, indoor smoking in private households or vehicles is legal in most countries, posing a great risk of exposure to second-hand smoke and the development of SHS-associated diseases [19]. Tobacco consumption inside cars is widespread. One or more windows are frequently opened to improve ventilation and diminish passive exposure. Nevertheless, PM exposure is increased even in vehicles with open windows [20, 21].

This study introduces a newly-designed, standardized approach for measuring PM concentrations (PM_10_, PM_2.5_, and PM_1_) inside a car cabin under different conditions without exposure of any person, posing a clear advantage over similar previous studies [20, 21]. This new platform is an improvement to the established platform of the Tobacco Smoke Particles and Indoor Air Quality (ToPIQ) as well as modified ToPIQ-2 studies [22, 23]. The aims of this study were (1) to mimic exposure of the car occupants and the driver to second-hand smoke emitted by a passenger in a standardized experimental setting and (2) to quantify the associated burden of particulate matter associated with this second-hand smoke.

## Methods

### Experimental setup

For measuring PM emissions of tobacco combustion products inside a car cabin, a Mitsubishi Space Runner 1991–1998 (syn. Mitsubishi Expo LRV) was parked in a garage at the Goethe-University of Frankfurt. The car had a total passenger and cargo volume of 3.709 m^3^ [24].

**Fig. 1:**
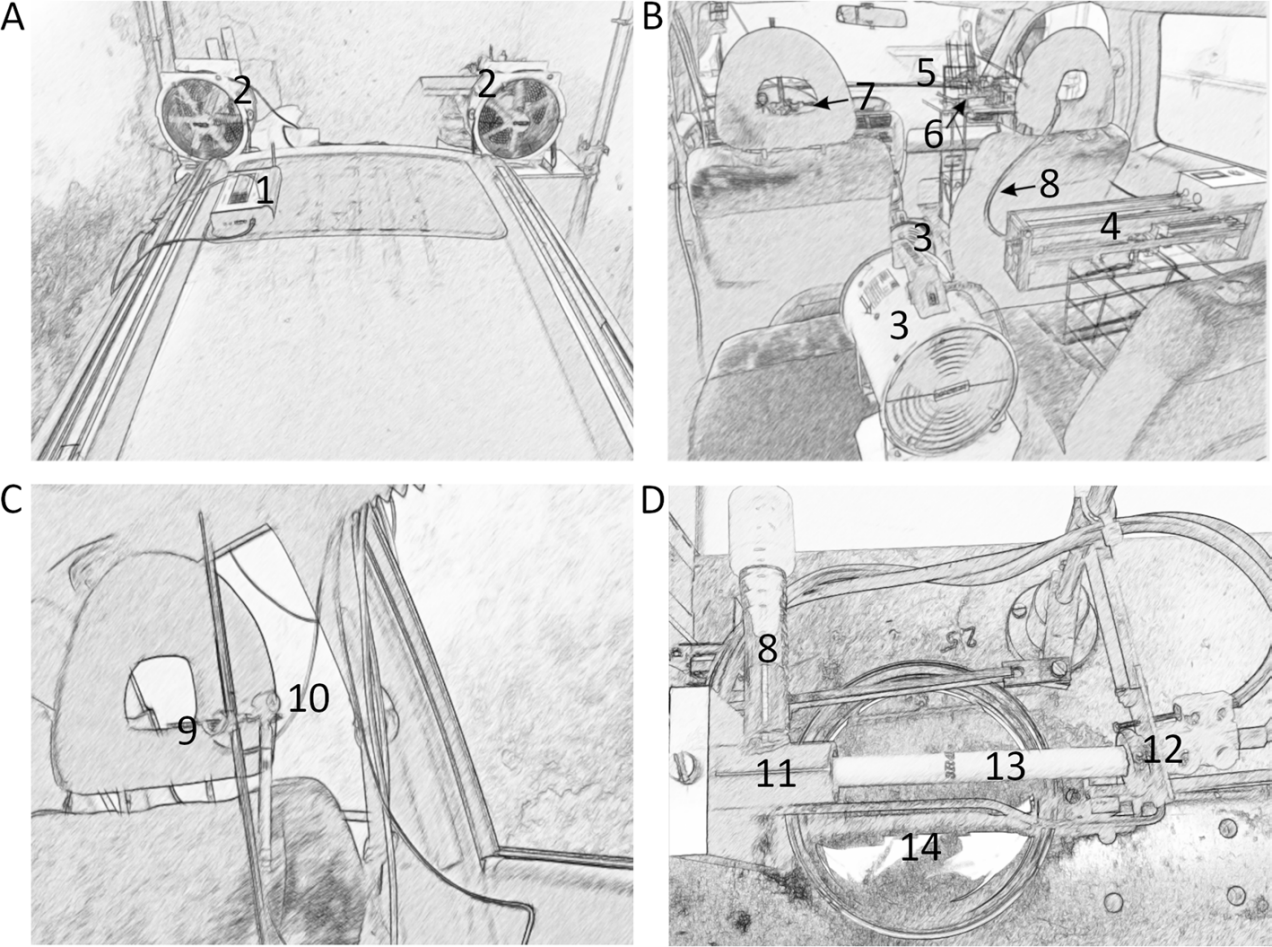
Measuring platform. Notes: **A** Outside. **B**-**D** Inside. A-1: Portable mini laser aerosol spectrometer (LAS, Grimm model 11-R). A-2: Fan (Model: Trotec TTV 4500 HP). B-3: Fan (Model: Master BML 4800). B-4: Automatic environmental tobacco smoke emitter (AETSE) B-5: Cigarette smoking device. B-6: Arrow marks the position of the cigarette. B-7: Arrow marks the suction point. B-8, D-8: Movable tube connecting the AETSE with the cigarette smoking device. C-9: Mobile tube connected to the LAS placed on the car roof (enables the LAS to suck interior vehicle air). C-10: Sensor for temperature, relative humidity, and air velocity (Grimm model 1.154). D-11: Cigarette mounting device. D-12: Automatic cigarette igniter. D-13: Cigarette. D-14: Petri dish filled with water

To avoid health risks caused by actively smoking tobacco, an automatic environmental tobacco smoke emitter (AETSE), equivalent to the ToPIQ-2 studies [23], was integrated into the car cabin. This machine was developed and constructed by Schimpf-Ing (Trondheim, Norway) and consisted of a smoke pump with a microcontroller unit that drove a stepper motor to move a plunger inside a 200 ml glass syringe [23]. The smoke pump was placed behind the passenger´s seat (Fig. 1B) and was connected to the cigarette smoking device via a polyamide tube (constructed by Daniel Müller, Institute of Occupational Medicine, Social Medicine and Environmental Medicine, Goethe University Frankfurt and Norbert Deffner, Workshop of physiology, University Hospital Frankfurt, Germany). The cigarette smoking device (simulating a smoking person) was placed on the passenger´s seat and was controlled by the microcontroller unit of the AETSE. The cigarettes were pushed manually into the mounting device holding the cigarette in a stable position (Fig. 1B, D). The cigarettes were lit by an automatic cigarette lighter. After the combustion phase, the cigarettes were expelled into a water bath to extinguish the cigarettes.

For air exchange, two fans (Model: Master BML 4800) were installed inside the car, one between the two front seats and one on the folded back rear seat next to the AETSE (Fig. 1B). For better ventilation of the car cabin, the researcher can open the tailgate and turn on the fans inside the car by remote control, thus blowing the cigarette smoke outside the garage. While cigarette combustion and ventilation of the vehicle, the researcher observes the experiment from a safe distance of approximately five meters outside the garage.

For future studies, two additional fans (Model: Trotec TTV 4500 HP) were pre-installed, one on each side of the front side windows outside the vehicle (Fig. 1A), producing a constant and reproducible airstream (airstream simulation). The fans have three power levels and can operate in three different modes. These modes can be distinguished by the airflow velocity in a distance of 1 m and include power level 1/3 with an airflow velocity of 0 km/h, power level 2/3 with an airflow velocity of 7.4 – 8.5 km/h, and power level 3/3 with an airflow velocity of 23 – 30.6 km/h. The Trotec TTV 4500 HP fans were not used in this study.

### Measuring system

PM measurements were carried out by a portable mini Laser Aerosol Spectrometer (LAS) Grimm Model 11-R. The LAS was positioned on the car top on the driver´s side (Fig. 1A). It can differentiate PM measurements into PM_10_, PM_2.5_, and PM_1_). Via a movable tube mounted on the passenger´s seat, 63 cm above the seating surface, the LAS can suck in interior vehicle air. This tube was positioned 70 cm away from the cigarette, 105 cm from the passenger´s side window, 34 cm from the driver´s side window, and 65 cm away from the steering wheel (Fig. 1C). Measurements were taken every 6 s. Temperature and relative humidity were measured at a device positioned 3 cm laterally to the tube at the same height. Only the on-board ventilation system that came with the vehicle was used for this experiment. It has four different power levels. For this study, only power level 2/4 was applied.

The SHS-dependent concentrations of PM inside the vehicle were measured under 4 different ventilation conditions. These comprised: *condition 1* (C1) all windows closed and the car ventilation was turned off, *condition 2* (C2), all windows closed and the car ventilation turned on power level 2/4, with the air directed towards the windshield*, condition 3* (C3), all windows closed and the car ventilation turned on power level 2/4, with the air directed towards the windshield and feet, and *condition 4 (*C4), all windows closed with the car ventilation turned on power level 2/4, with the air directed towards body and head.

### Tobacco products

The 3R4F reference cigarettes used in this study yield an average of 9.4 mg tar, 0.73 mg nicotine and 12 mg carbon monoxide [25].

### Smoking protocol

All cigarettes tested followed the same smoking protocol. Automatic ignition of the cigarette by the cigarette smoking device was followed by two initial puffs. The interval between the two initial puffs was one second. Two subsequent cigarette puffs per minute with a time interval of 30 s were taken. Each puff had a duration of 3 s and a volume of 40 ml. After a total of 10 puffs, the cigarette was expelled from the cigarette smoking device and extinguished in a petri dish filled with water. The cigarette smoking device was restarted after 5 min of extensive full vehicle ventilation. Each condition was repeated 24 times, accounting for a total number of 96 smoked cigarettes. Combustion of cigarettes that extinguished prematurely had to be repeated. They were not considered for the subsequent data processing.

### Data processing and analysis

Each measuring period was divided into three intervals. The *first interval* started with a baseline PM measurement which was acquired after thorough vehicle ventilation for at least five minutes after each smoked cigarette. The *second interval* consisted of the time between ignition and extinction of the cigarette lasting for 4.5 min (combustion phase). The *third interval* lasted for at least 5.5 min and represents the phase of post-combustion. Every 6 s, the LAS measures PM_10_, PM_2.5_, and PM_1_ concentrations. Each evaluated cigarette dataset consists of 101 single measurements resulting in exactly 10 min.

PM_10_, PM_2.5_, and PM_1_ are average concentrations after a given period of time. They are measured after 4.5 min (1. – 46. measurement) and after 10 min. (1 – 101. measurement). PM peaks represent single values of measurements at 4.5 min (46. measurement) and at 10 min (101. measurement).

### Statistical analysis

Statistical evaluation of data was performed by using Prism version 6 (GraphPad Software, La Jolla California, USA, www.graphpad.com). Shapiro–Wilk, D`Agostino-Pearson, and Kolmogorov–Smirnov tests were used to determine standard distribution (passed). A one-way analysis of variance (ANOVA) with Tukey’s multiple comparison test followed. Level of significance was set at *p* = 0.05.

## Results

The baseline PM_10_, PM_2.5_, and PM_1_ after a ventilation interval of at least 5 min were 30.6 ± 11.5 µg/m^3^, 27.9 ± 11 µg/m^3^, and 24.6 ± 11.2 µg/m^3^, respectively.

PM_10_, PM_2.5_, and PM_1_ mean concentrations (C_mean_) after 4.5 min, smoked without any ventilation (C1), were significantly higher (*p* < 0.0001) than PM concentrations with the three other ventilation conditions (C2 – C4). C_mean_ PM_10_ during experimental conditions C2 – C4 was 47.5 to 58.4% lower compared to C1. PM_2.5_ and PM_1_ at C2 – C4 were 39.6 – 58% lower than at C1 (Table 1). No significant difference could be seen comparing C2 – C4 (*p* = 0.0752 – 0.9999). Nevertheless, C4 presented slightly higher PM C_mean_ than C2 and C3 after 4.5 min.

**Table 1:** Mean concentrations (A) and peak emissions (B) of particulate matter (PM_10_, PM_2.5_, PM_1_)

Condition	Minutes after ignition	PM_10_ (µg/m^3^)	PM_2.5_ (µg/m^3^)	PM_1_ (µg/m^3^)
**C1**	4.5	A: 1150 ± 462 B: 2207 ± 1294	A: 1132 ± 452 B: 2166 ± 1251	A: 861.6 ± 271 B: 1421 ± 516
	10	A: 1608 ± 461 B: 1989 ± 438	A: 1583 ± 451 B: 1959 ± 428	A: 1133 ± 245.9 B: 1375 ± 214
**C2**	4.5	A: 478.8 ± 37 − 58.4% B: 630.2 ± 45.5 − 71.5%	A: 475.2 ± 36 − 58% B: 624.6 ± 44.7 − 71.2%	A: 428.7 ± 246 − 50.2% B: 543.2 ± 32.4 − 61.8%
	10	A: 412.4 ± 30.2 − 74.4% B: 129.8 ± 13.4 − 93.5%	A: 409.5 ± 29.9 − 74.1% B: 129.4 ± 13.5 − 93.4%	A: 369.3 ± 24.8 − 67.4% B: 123.9 ± 13.1 − 91%
**C3**	4.5	A: 489.8 ± 71.2 − 57.4% B: 667.2 ± 129 − 69.8%	A: 485.2 ± 70.1 − 57.1% B: 660.3 ± 126 − 69.5%	A: 430.2 ± 53.3 − 50.1% B: 558.9 ± 91.8 − 60.7%
	10	A: 410.6 ± 62.4 − 74.5% B: 124 ± 24.8 − 93.8%	A: 407.1 ± 61.5 − 74.3% B: 123.7 ± 24.7 − 93.7%	A: 362.1 ± 48.7 − 68% B: 118.2 ± 22.6 − 91.4%
**C4**	4.5	A: 603.7 ± 59.9 − 47.5% B: 845.3 ± 203 − 61.7%	A: 597.4 ± 58.4 − 47.2% B: 836 ± 199 − 61.4%	A: 520.3 ± 38.6 − 39.6% B: 692.5 ± 133 − 51.3%
	10	A: 469.9 ± 45.6 − 70.8% B: 125.6 ± 17 − 93.7%	A: 465.5 ± 44.5 − 70.6% B: 125.2 ± 16.9 − 93.6%	A: 407.7 ± 30.2 − 64% B: 118.2 ± 15.6 − 91.4%

**Notes:** Mean concentrations (C_mean_) and peak emissions of PM_10_, PM_2.5_, and PM_1_ with given standard deviation (SD) of 3R4F reference cigarettes under four different conditions (C1 – C4). Deviation of C_mean_ PM and PM peaks from 3R4F reference cigarette C1 in percentage after 4.5 min and 10 min, respectively. Condition 1 (C1): All windows closed, and the car ventilation turned off. Condition 2 (C2): All windows closed, and the car ventilation turned on power level 2/4, with air directed towards the windshield. Condition 3 (C3): All windows closed, and the car ventilation turned on power level 2/4, with the air directed towards the windshield and feet. Condition 4 (C4): All windows closed, and the car ventilation turned on power level 2/4, with the air directed towards body and head. A: C_mean_ after 4.5 min and 10 min. B: Mean peak emissions at 4.5 min and 10 min.

PM C_mean_ after 10 min under C1 were significantly higher (*p* < 0.0001) than those smoked under C2 – C4. The PM_10_ mean value of cigarettes smoked under C1 was 290% higher than under C2. PM_2.5_ and PM_1_ mean values under C1 were 287% and 207% higher than their counterparts of C2 (Table 1).

**Fig. 2:**
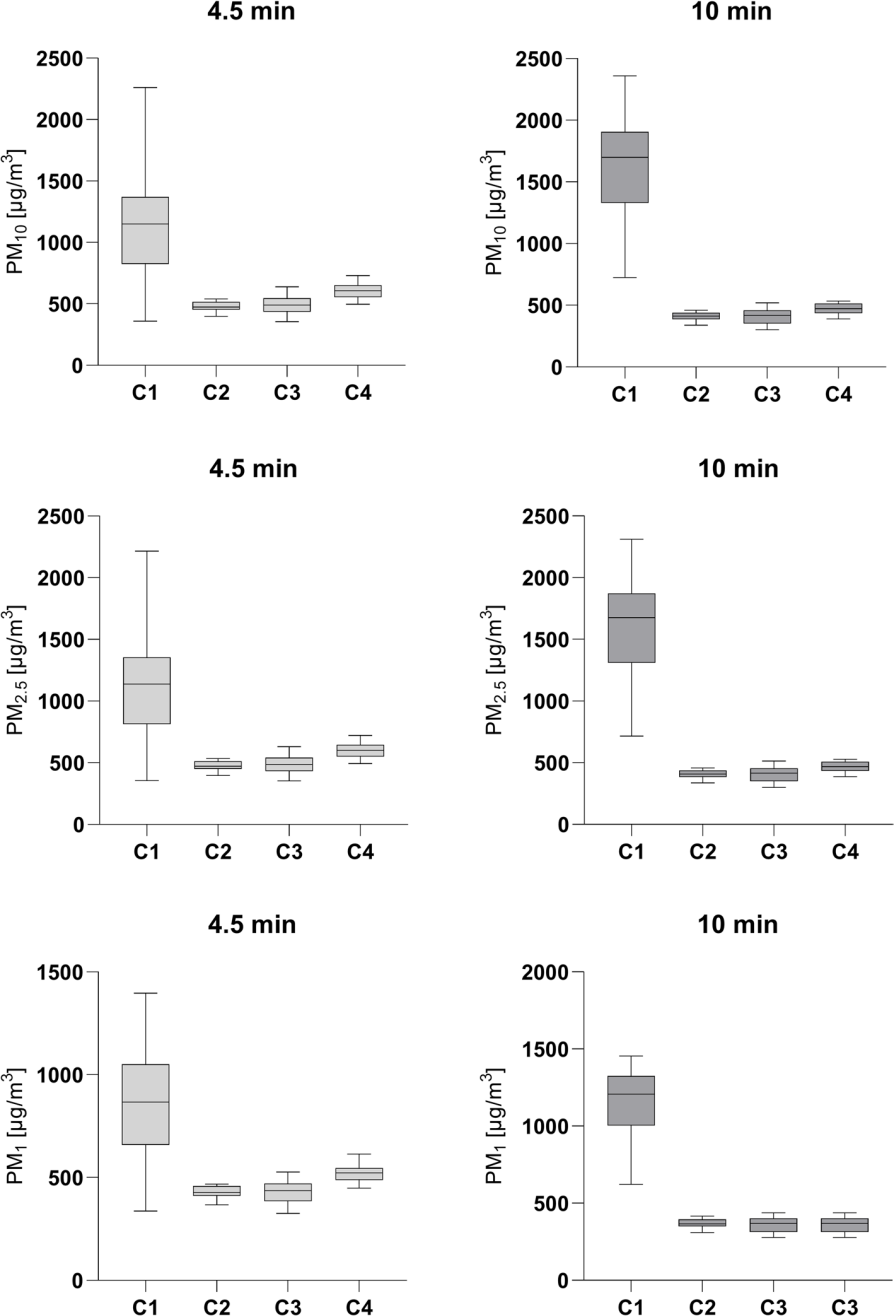
Boxplots (min to max whiskers) graphically display data seen in Table 1. Notes: PM: Particulate matter. Condition 1 (C1): All windows closed, and the car ventilation turned off. Condition 2 (C2): All windows closed, and the car ventilation turned on power level 2/4, with air directed towards the windshield. Condition 3 (C3): All windows closed, and the car ventilation turned on power level 2/4, with the air directed towards the windshield and feet. Condition 4 (C4): All windows closed, and the car ventilation turned on power level 2/4, with the air directed towards body and head

The highest PM mean values showed the 3R4F reference cigarette without ventilation (Fig. 2). 3R4F reference cigarettes of C2 – C4 showed similar PM mean values. Here, the largest difference could be observed comparing PM_10_ of C3 and C4. Directing the ventilation towards the body and head (C4) led to a 14% increase of PM_10_ compared to C3 (ventilation towards windshield and feet). Although different ventilation directions (C2 – C4) did not show any significant differences in PM_10,_ PM_2.5_, and PM_1_ (*p* = 0.5460 – 0.9999), we saw a trend regarding the PM values measured during C4 that were slightly higher than those of C2 and C3 after 10 min.

PM peaks at 4.5 min and 10 min were the highest during ventilation condition C1. PM peaks at 4.5 min and 10 min showed significant differences (*p* < 0.0001) when ventilation condition C1 was compared with conditions C2 – C4. No significant differences could be seen comparing PM peaks of C2 – C4 at 4.5 min (*p* = 0.2191 – 0.9973) with respective PM peaks at 10 min (*p* = 0.9977 – 0.9999).

PM_10_, PM_2.5_, and PM_1_ decreased from the baseline by 9.9%, 9.6%, and 3.2% respectively, comparing the peaks at 4.5 min and 10 min. PM peaks at 4.5 min decreased by up to—71.5% comparing C1 with conditions C2 – C4. After 10 min, a maximum PM reduction of 93.8% was seen comparing C1 and conditions C2 – C4.

**Fig. 3:**
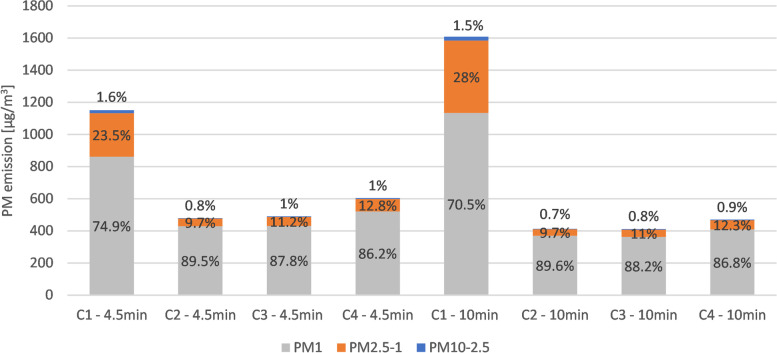
Distribution pattern of mean concentrations PM_10-2.5_, PM_2.5–1_, and PM_1_ after 4.5 min and 10 min. Notes: PM was generated by smoking cigarettes in a compact car. PM: Particulate matter. Condition 1 (C1): All windows closed, and the car ventilation turned off. Condition 2 (C2): All windows closed, and the car ventilation turned on power level 2/4, with air directed towards the windshield. Condition 3 (C3): All windows closed, and the car ventilation turned on power level 2/4, with the air directed towards the windshield and feet. Condition 4 (C4): All windows closed, and the car ventilation turned on power level 2/4, with the air directed towards body and head

Particle distribution is illustrated in Fig. 3. Looking at C_mean_ of measurement ranges, PM_1_ accounted for 74.9% of total PM at 4.5 min and 70.5% at 10 min emitted under C1. PM_2.5–1_ made up 23.5%, and 28%, while PM_10-2.5_ only accounted for 1.5 – 1.6% of PM emissions under C1. Condition 2 showed the highest amount of PM_1_ with 89.5% of total PM, closely followed by C3 (88.2% of total PM) and C4 (86.8% of total PM). PM_2.5–1_ of C2-C4 varied between 9.7 – 12.8% of total PM, while PM_10-2.5_ made up only 0.70 – 1%. Only minor variations could be seen comparing PM_10_, PM_2.5_, and PM_1_ emissions after 4.5 min and 10 min.

At all PM measurements, the temperature inside the car cabin varied between 17 – 21 °C. Relative humidity inside the cabin was between 42—55%.

## Discussion

The presented study describes a newly developed experimental approach to quantify PM emissions of second-hand smoke in a vehicle. The data demonstrate that in-vehicle smoking generates harmful particle emissions of varying diameters even with the vehicle ventilation system turned on. If the experimental setting mimicked a scenario with no ventilation initiated by the car occupants, the particle load was significantly higher when the ventilation was started, albeit the differences in particle load exposure between the ventilation conditions were minimal.

Prior studies have investigated tobacco smoke pollution in cars under in vivo conditions exposing human smokers [20, 21, 26–31]. Thus, acquired data of PM_2.5_ by Sendzik et al. under similar ventilation conditions resulted in comparable PM concentration levels as in this study [21]. There are important ethical concerns regarding the exposure of humans to toxic tobacco smoke for research purposes. Therefore, the strength of this study is to introduce a new mechanical system that allows research on secondhand tobacco smoke to be conducted without exposing the researchers or any other person to tobacco smoke. The highly standardized smoking procedure ensures optimal data acquisition and comparison. During conditions C2—C4, the on-board ventilation system of the car was kept on power level 2/4 as we considered it a realistic setting for drivers to use. Although temperature and relative humidity may impact PM concentrations, they were intentionally left uncontrolled, which might seem as a limitation of this study. This experimental approach was chosen because it imitates real-life driving conditions in a car without air-conditioning or heat [32, 33]. As mentioned in previous ToPIQ studies, the AETSE cannot exactly imitate a real smoker [22, 23, 34, 35]. Therefore, it was surprising that the PM data could be compared with data originating from research using human smokers. In contrast to ToPIQ I, the ToPIQ II study design used a larger smoking chamber (2.88 m^3^), thus being more comparable to the interior car volume of the Mitsubishi Space Runner (3.709m^3^) [23, 24]. Due to the larger interior volume of the car, the reference cigarettes PM_2.5_ exposure measured in the ToPIQ II study by Gerber et al. is about 9% higher than the corresponding PM values presented in this study [23]. The influence of varying interior volumes on PM burden should be the focus of future studies.

The findings of extremely high PM values after 10 min of cigarette smoking without any ventilation were alarming. Further, the 3R4F reference cigarettes smoked under diverse ventilation conditions displayed similarly high PM levels (no significance, *p* > 0.05). The mean concentrations of PM were 3—4 times less compared to cigarettes smoked without ventilation. Under C3, PM_10_, PM_2.5_, and PM_1_ decreased by 74.5%, 74.3%, and 68%, respectively, after 10 min compared to C1, representing the highest reduction of PM for the investigated ventilation conditions. On the contrary, the lowest decrease of PM after 10 min was measured under C4 (Table 1).

Even after 10 min, the measured C_mean_ of PM_10_ (> 400 µg/m^3^) had exceeded the WHO 24 h threshold by more than factor 9. PM_10_ concentrations of cigarettes smoked without ventilation were 35 times higher than the recommended WHO threshold for PM_10_ [8]. Moreover, these high PM concentrations drastically exceeded the measurements conducted by Dröge et al. (traffic PM measurements inside a driving vehicle cabin, among others, with closed windows) showing PM_2.5_ values of 5.2 – 23.2 µg/m^3^ and PM_1_ values that ranged from 4.9 – 22.6 µg/m^3^ [36]. The higher C_mean_ values after 10 min compared to C_mean_ after 4.5 min under C1 is due to the sustained high plateau concentration after the cigarette has been extinguished. The reduction of PM concentrations from 4.5 min to 10 min by 13.8 – 22.2% under C2 – C4 demonstrates the effect of in-vehicle ventilation during a smoking session. Ventilation dilutes the in-cabin air with ambient air, thus increasing the air exchange rate and decreasing the PM concentration [31, 37]. It is not yet known whether the ventilation produces an airstream that pushes PM into the back of the vehicle, thereby increasing the PM concentration where children are usually seated. Experiments have already indicated that smoking a cigarette with an opened window does not decrease the PM exposure in the back seat [38]. Although smoking with one or more opened windows increases the air exchange rate, the SHS exposure is still highly elevated [20, 38–40]. Schober et al. compared the PM emissions of IQOS, E-Cigarettes and tobacco cigarettes under six different ventilation conditions in various cars with varying interior volumes [41]. Combustion of tobacco cigarettes reached higher emissions of PM_2.5_ (64—1988 μg/m^3^) than IQOS or E-Cigarettes. Compared to our investigation (PM_2.5_: 407—1583 μg/m^3^), their PM_2.5_ emission range is wider due to different experimental conditions [41]. Sohn et al. measured the PM emissions of cigarettes under three different ventilation conditions [20]. Similar to our study, they divided the measurements into three phases: The pre-smoking phase, smoking phase, and post-smoking phase. While they concluded that the PM_2.5_ concentration exceeded the US National Ambient Air Quality Standard of 35 µg/m^3^_,_ their investigation lacks differentiation of PM_10_ and PM_1_ emissions. Neither of the two aforementioned study designs investigated the effect of the on-board ventilation system on PM emissions as presented in our research model [20, 41].

Figure 3 differentiates PM into PM_10-2.5_, PM_2.5–1_, and PM_1_, thus comparing the individual mass of different particle sizes created through cigarette combustion. After 10 min 70.5—89.6% of the total PM mass is ≤ 1 µm, while PM_2.5–1_ accounts for 9.7—28% and PM_10-2.5_ for 0.7—1.5%. Due to gravitational settling, coarse particles have faster deposition rates than fine particles [42, 43]. Nevertheless, the deposition rate of PM is highly variable and depends on many factors (e.g., humidity, temperature, air turbulence, surface roughness, thermophoresis, turbophoresis, spatial distribution, electrostatic effects) [33, 44]. In contrast to the PM fractions PM_2.5–1_ and PM_1_, PM_10-2.5_ is more impacted by gravity [33]. Therefore, the high concentration of fine particles after 10 min is due to slow gravitational sedimentation and high fine particle generation during cigarette combustion [7, 42, 45]. The portion of PM_1_ was 16.3 – 19.1% higher for C2 – C4 compared to C1 after 10 min. That is likely caused by the ventilation, creating air turbulence leading to slower gravitational sedimentation of fine particles [33].

A high concentration of small particles < 2.5 µm is particularly alarming, as they can penetrate deeply into the respiratory system causing severe health burdens [46]. Children exposed to small particles are especially vulnerable and can develop various diseases (asthma, cancer, decreased lung function, otitis, neurobehavioral problems, etc.) [47, 48]. In 2010, additional health care services for US children aged 3–14 of 62.9 million dollars were linked to preventable SHS [49]. An observational study carried out in Italy showed that children were exposed to in-cabin SHS in 0.9% of passing by vehicles [50]. Therefore, children should be protected from SHS by law, prohibiting in-door (in-vehicle) smoking.

The on-board ventilation system (C2 – C4) drastically reduced the PM peaks after 4.5 min and 10 min. Compared to C1, it decreased PM_10_, PM_2.5_, and PM_1_ peaks at 4.5 min by 61.7—71.5%, 61.4—71.2%, 51.3—61.8%, and at 10 min by 93.5 – 93.8%, 93.4 – 93.7%, and 91 – 91.4%, respectively (Table 1). Therefore, the majority of PM concentration peaks (> 61%) is reduced at the end of the second interval (after 4.5 min).

In 2019 Campagnolo et al. accurately showed a correlation of PM concentration inside a car cabin depending on the emission standard of the car driving ahead. New emission standard cars (Euro 6) generate 34% less PM_0.3–1_ for the following car than compared to its older predecessors (i.e., Euro 0–2) [51]. This study presents a great example of the benefit of strict PM emission laws for vehicles.

The new measuring platform poses multiple opportunities for future investigations. PM exposure under diverse ventilation scenarios with different degrees of window openings is of the highest interest. The two outside fans can be used at different power levels to simulate an airstream around a moving vehicle. Measurements during this simulation may add important data about PM exposure during a car drive with opened windows. The influence of air conditioning could be investigated in upcoming studies. The cigarette smoking device can aid in investigating the effects of chain-smoking on PM concentration in a vehicle. Positioning multiple LAS at different locations inside the car cabin could generate important data about the distribution of SHS inside the car cabin under various ventilation conditions simultaneously.

## Conclusion

The presented new platform enables researchers to safely measure PM emissions from tobacco products in a car cabin without exposure to SHS. Investigating and comparing the effects of multiple different ventilation scenarios on PM concentrations is important for future studies. This study demonstrates the vast PM burden created by smoking in vehicles and shows the importance of banning smoking in cars. We hope that investigations carried out may aid in encouraging governments and people to create a smoke-free world.

## Data Availability

The datasets used and/or analyzed during the current study are available from the corresponding author on reasonable request.

## Abbreviations

AETSE: Automatic environmental tobacco smoke emitter
ANOVA: Analysis of variance
C1: All windows closed, and the car ventilation turned off
C2: All windows closed, and the car ventilation turned on power level 2/4, with air directed towards the windshield
C3: All windows closed, and the car ventilation turned on power level 2/4, with the air directed towards the windshield and feet
C4: All windows closed, and the car ventilation turned on power level 2/4, with the air directed towards body and head
C_mean_: Mean concentration
LAS: Laser aerosol spectrometer
p: Probability value
PM: Particulate matter
SHS: Second-hand smoke
ToPIQ: Tobacco Smoke Particles and Indoor Air Quality
WHO: World Health Organization
3R4F: 3R4F reference cigarettes
